# Design and Applications of Biodegradable Polyester Tissue Scaffolds Based on Endogenous Monomers Found in Human Metabolism

**DOI:** 10.3390/molecules14104022

**Published:** 2009-10-12

**Authors:** Devin G. Barrett, Muhammad N. Yousaf

**Affiliations:** Department of Chemistry and Carolina Center for Genome Science, University of North Carolina at Chapel Hill, Chapel Hill, NC 27599-3290, USA

**Keywords:** biodegradable, polyester, biocompatible, elastomer, tissue scaffold

## Abstract

Synthetic polyesters have deeply impacted various biomedical and engineering fields, such as tissue scaffolding and therapeutic delivery. Currently, many applications involving polyesters are being explored with polymers derived from monomers that are endogenous to the human metabolism. Examples of these monomers include glycerol, xylitol, sorbitol, and lactic, sebacic, citric, succinic, α-ketoglutaric, and fumaric acids. In terms of mechanical versatility, crystallinity, hydrophobicity, and biocompatibility, polyesters synthesized partially or completely from these monomers can display a wide range of properties. The flexibility in these macromolecular properties allows for materials to be tailored according to the needs of a particular application. Along with the presence of natural monomers that allows for a high probability of biocompatibility, there is also an added benefit that this class of polyesters is more environmentally friendly than many other materials used in biomedical engineering. While the selection of monomers may be limited by nature, these polymers have produced or have the potential to produce an enormous number of successes *in vitro* and *in vivo*.

## Introduction

The human body is a complex network of different tissues and organs that all have specific functions. Tissue engineering has emerged as a route for repairing or replacing the injured tissue with designed scaffolds when these tissues are damaged and the body cannot heal them [1,2,3]. Recently, research has suggested that scaffolds for tissue engineering should mimic the mechanical properties of the natural tissues. Throughout the body, there are many diverse biological materials that demonstrate unique sets of mechanical characteristics [4,5,6,7,8,9]. For example, heart valves, bladder, smooth muscle, collagen fibers, and elastin all exhibit mechanical properties that differ over several orders of magnitude (Table 1). Therefore, much research has been devoted to designing biocompatible scaffolds that extend over a wide range of rigidities, flexibilities, and strengths. One such class of materials that has demonstrated this mechanical versatility is polyesters. These polymers are biodegradable and have thus gained popularity as potential tissue scaffolds [10,11]. Additionally, polyester elastomers often exhibit degradation rates that can be easily tuned to meet the needs of a particular application. 

**Table 1:** Mechanical properties of some soft tissues and biodegradable tissue scaffolds.

Material	Modulus (MPa)	Ult. Stress (MPa)	Ult. Strain (%)	Ref.
Bovine elastin	1.1	2	-	4
Collagen fibers	100	-	50	5
Knee articular cartilage	2.1-11.8			6,7
Smooth muscle (relaxed)	0.006	-	300	5
Smooth muscle (contracted)	0.01	-	300	5
Human bladder	0.25	0.27	0.69	8
Porcine bladder	0.26	0.32	1.66	8
Porcine aortic heart valve	6.4 – 44.7	1.4–8.3	48.7–134.8	9
PGA^a^	6,900	70	< 3	2,82
PLLA^b^	1,200–2,700	28 - 50	6	2,82
PDLLA^c^	1,900–2,400	29 - 35	6	2,82
PCL^d^	0.21–0.34	20.7	300–500	2
PTMC^e^	6.3–6.8	12 - 24	820–831	90
PLGA^f^	1.4–2.8	41.4–55.2	3–10	2
PLCL^g^	0.192–68.573	0.57–8.55	175–854.4	83,84,85
PLTMC^h^	4–1,900	1 - 53	4–830	88,89,92
PGS^i^	0.282	> 0.5	> 267	34,93,94
PGSA^j^	0.048 - 1.375	0.054 - 0.498	47.4–170	29,95,96
PPS^k^	0.37 - 378.0	0.57 - 17.64	10.90–205.2	97,98
APS^l^	2.45 - 4.24	1.33 - 1.69	64–92	99
PDC^m^	1.60–13.98	2.93–11.15	117–502	101,108
PGlSu^n^	-	-	-	118
PTK^o^	0.1–657.4	0.2–30.8	22–583	135
PPF^p^	0.9–4,500	.05–120	5 -20	138,139,140,141,142

^a^– poly(glycolide); ^b^ – poly(l-lactide); ^c^ – poly(d,l-lactide); ^d^ – poly(caprolactone); ^e^ – poly(trimethylene carbonate); ^f^ – poly(lactide-co-glycolide); ^g^ – poly(lactide-co-caprolactone); ^h^ – poly(lactide-co-trimethylene carbonate); ^i^ – poly(glycerol sebacate); ^j^ – poly(glycerol sebacate acrylate); ^k^ – poly(polyol sebacate); ^l^ – poly(1,3-diamino-2-hydroxypropane-*co*-polyol sebacate); ^m^ – poly(diol-co-citrate); ^n^ – poly(glycerol succinate); ^o^ – poly(triol α-ketoglutarate); ^o^ – poly(propylene fumarate).

In the last decade, as synthetic polyester elastomers have become very popular options for soft tissue engineering, research has explored a wide range of scaffolds that have mechanical properties that match those of the natural tissue [6,7,12,13,14,15]. The rigidity and flexibility of an underlying scaffold has been shown to enhance cellular behavior and/or tissue viability [16,17,18,19,20]. Within the class of polyester scaffolds, many examples of both thermoplastics and thermosets have been synthesized [21,22,23,24,25,26,27,28,29,30]. When compared to thermoplastic materials, thermosets offer a number of advantages for biomedical applications. For example, thermoplastic elastomers often contain crystalline regions, which cause heterogeneous degradation and a non-predictable loss of mechanical strength [31,32]. The three-dimensional (3D) geometry of a thermoplastic material is also commonly lost during the hydrolysis period [33,34]. On the contrary, thermosets can be designed from amorphous precursors, allowing for consistent degradation rates and a linear loss of mechanical strength [11,34]. Polyester thermosets also often degrade by surface erosion, resulting in the retention of the 3D structure throughout the hydrolysis period [34].

Synthetic polyester elastomers have been designed to incorporate a wide range of monomers. With the requisite of biocompatibility, some polymers have been based on small molecules that are endogenous to the human metabolism. Although the use of metabolite monomers is not required for biocompatibility, the use of molecules that are normally present in the human metabolism offers a route to minimize toxic side effects. Some of these polyesters, during degradation, release molecules that the body can resorb, metabolizing them in various physiological pathways. This review will focus on novel polyester elastomers composed of at least one monomer endogenous to the human metabolism. While examples of thermoplastics are described, this review will focus primarily on polyester thermosets.

## Biological Role of Common Polyester Monomers

### Lactic Acid

During exercise, activity leads to glycogenolysis, the breakdown of glycogen in the muscles, which generates ATP as a source of energy [35,36,37,38,39]. Initially, glycolysis results in the production of pyruvate, which can enter the Krebs cycle and ultimately produce ATP through the electron transport chain. However, as muscular activity continues, a lack of oxygen prevents the use of this pathway. The Krebs cycle stalls, causing pyruvate to accumulate. By entering the Cori cycle, pyruvate is converted to lactate by lactate dehydrogenase [40,41,42,43,44,45]. The liver then receives the lactate, converts it to pyruvate, which is in turn converted to glucose.

### Glycerol

Glycerol is essential to multiple vital metabolic pathways inside cells. For example, glycerol forms the backbone of many fats, including triglycerides, formed from the condensation of glycerol and three fatty acids through ester bonds, and phospholipids, which are composed of a diglyceride, a phosphate group, and a polar molecule [46,47,48,49,50,51,52]. Most of the fats digested by humans are composed of triglycerides, which cannot be absorbed by the duodenum. Pancreatic lipase is able to hydrolyze the ester bonds, forming glycerol, fatty acids, monoglycerides, or diglycerides, all of which are much more easily absorbed. Phospholipids, on the other hand, are crucial to compartmentalizing the interior of cells. With hydrophobic and hydrophilic moieties, these amphiphilic molecules self-assemble to create the lipid bilayers that form the majority of cell membranes.

Glycerol can also take part in glycolysis or gluconeogenesis, depending on cellular conditions [53,54,55,56,57]. Glycerol kinase is able to phosphorylate glycerol, forming glycerol-3-phosphate. After being oxidized, the product is dihydroxyacetone phosphate, which is an isomer of glyceraldehyde-3-phosphate. During gluconeogenesis, fructose-1,6-bisphosphate can be formed by the condensation of glyceraldehyde-3-phosphate and dihydroxyacetone phosphate, eventually leading to the biosynthesis of pyruvate and acetyl coenzyme A (CoA). Glyceraldehyde-3-phosphate can also continue through the glycolysis metabolic pathway.

**Figure 1:**
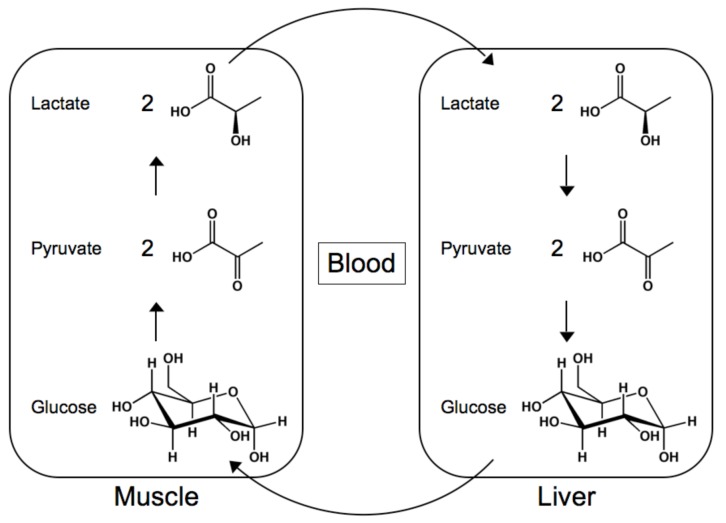
The structures and reactions of the Cori cycle.

### Sebacic Acid

Sebacic acid is an α,ω-diacid that is ten carbons in length. It is a natural intermediate in the metabolism of medium- and long-chain α-carboxylic acids [58,59,60,61]. *In vivo*, dicarboxylic acids are formed by the ω-oxidation of α-carboxylic acids. Because it is an even-chain diacid, it can be completely metabolized to form acetyl (CoA) and succinyl CoA. Acetyl CoA is crucial to the Krebs cycle and the formation of isoprenoids, while succinyl CoA is an intermediate in the Krebs cycle and a key component of porphyrin biosynthesis.

### Sugar Alcohols

Sugar alcohols are the reduced form of aldoses and ketoses. They are commonly used as sugar substitutes due to their lower caloric content [62,63,64,65]. This class of polyols, which includes glycerol, is not usually completely absorbed through the small intestine into the blood stream. These properties make sugar alcohols a popular sweetener for diabetics due to digestion resulting in a smaller change in blood glucose levels when compared to sucrose. Xylulose is converted into xylitol by l-xylulose reductase during mammalian carbohydrate metabolism [66,67,68,69]. Similarly, sorbitol, the sugar alcohol form of glucose, is converted to fructose by sorbitol dehydrogenase [70,71,72,73].

### Citric, Succinic, and α-Ketoglutaric Acids

Citric acid, succinic acid, and α-ketoglutaric acid (αKA) are key intermediates in the Krebs cycle (as known as the citric acid cycle) [74,75]. The Krebs cycle begins with the production of the citrate, the deprotonated form of citric acid. Acetyl CoA is hydrolyzed in the presence of oxaloacetate and citrate synthase, resulting in citrate. The enzyme aconitase then dehydrates and rehydrates citrate, forming isocitrate. Oxidation of isocitrate by isocitrate dehydrogenase produces oxalosuccinate, which rapidly degrades into carbon dioxide and αKA. α-Ketoglutarate dehydrogenase then couples αKA to acetyl CoA through oxidative decarboxylation, forming succinyl CoA. Succinate is then synthesized from the hydrolysis of succinyl CoA by succinyl CoA synthetase. Succinate dehydrogenase then converts succinate to fumarate, simultaneously reducing flavin adenine dinucleotide (FAD) to FADH_2_. Fumarate is converted to L-malate, which is oxidized into oxaloacetate. Oxaloacetate is then involved in the initial step of the Krebs cycle, the production of citrate.

**Figure 2:**
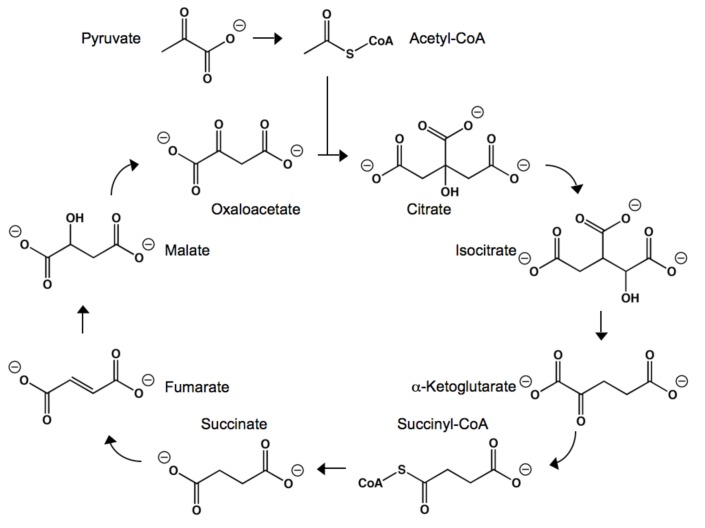
The structures and reactions involved in the Krebs cycle.

Outside of the Krebs cycle, α-ketoglutarate is also involved in the biosynthesis of amino acids [76,77,78,79]. Through transamination, the amino group of certain amino acids can be transferred to αKA, often producing molecules important to other metabolic pathways. For example, when catalyzed by a transaminase, alanine and αKA can be converted to glutamate and pyruvate, while aspartate and αKA can react to form glutamate and oxaloacetate.

## Polyesters as Tissue Scaffolds

### Poly(d,l-lactide-*co*-ε-caprolactone)

The Shin’oka group has published several manuscripts describing their tissue engineering work in human patients [80,81,82]. Motivated by both the negative side effects and reduced growth potential of prosthetic materials, they have focused on using biodegradable materials. Initially, a poly(lactide-co-ε-caprolactone) co-polymer (PLCL) was reinforced with poly(lactide) (PLA) or poly(glycolide) (PGA) [83]. PLCL has been used by other groups for tissue engineering, although with much less clinical data [84,85,86]. The devices were designed by fabricating tubular biodegradable scaffolds that can serve as a conduit between functional sections of the pulmonary artery.

**Figure 3:**
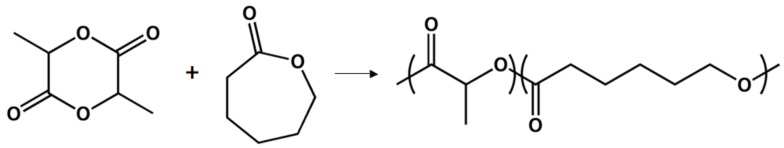
Poly(lactide-co-caprolactone) resulting from the ring-opening polymerization of the corresponding monomers.

Two different cell types were seeded onto the polyester scaffolds. First, segments of saphenous veins were surgically removed and used as a source of cells. A week before the surgery, cells were added to the scaffold and maintained in culture medium. This surgical option was performed on two patients. As a time-sensitive alternative, bone marrow cells (BMCs) were aspirated from the superior iliac spine as cardiac surgery began. After seeding BMCs onto scaffolds, the artificial pulmonary artery segments were kept in culture medium for 2–4 h before use. After November of 2001, the second strategy was used exclusively, due to the fact that culturing vein cells can lend itself to infections and an insufficient amount of cells. The strategy involving BMCs was expected to be successful because, as previously reported, endothelialization was observed after seeding BMCs onto the surface of a synthetic graft in dog models [87,88]. This strategy was applied to 22 patients, with chest radiographs showing excellent recovery and no post-operative complications or obstructions observed at the area of the implant. 

In fact, follow-up examinations were conducted 490 ± 276 days after the original surgeries [82]. No complications such as stenosis, obstruction of autografts, or thrombosis were noticed. In addition, the autografts were able to increase in diameter as a function of time. At the time of follow-up, the average tube diameter was 110 ± 7% of the original size. These results indicate that this may be an appropriate alternative to prosthetic materials in pediatric patients due to the growth potential. This innovative work from the Shin’oka research group, including a large amount of clinical data, has demonstrated that vascular autografts designed by seeding BMCs onto polyester scaffolds provided an ideal alternative to prosthetic materials during cardiac surgery.

### Poly(d,l-lactide-*co*-trimethylene carbonate)

The Feijen lab has extensively studied the use of 1,3-trimethylene carbonate (TMC) and d,l-lactide (DLLA) as tissue scaffolds [89,90]. Porous and biodegradable scaffolds were prepared from amorphous elastomeric co-polymers of TMC and DLLA to be used in heart tissue engineering. While poly(TMC) is not an ideal material for tissue scaffolds, TMC-DLLA co-polymers with either 20 or 50 molar percentage of TMC are completely amorphous and flexible, making them ideal for the preparation of scaffolds for soft tissue scaffolds [91]. Using a salt leaching technique, highly porous, compression-molded poly(ester carbonate) films were prepared. Adjusting the size of the salt particles and the polymer-to-particle weight ratio in the composite preparation controlled the pore size and the porosity of the scaffold, respectively. The poly(ester carbonate) films were also observed during degradation in PBS at 37 °C. The size of the films and the molecular weight of the polymer chains both gradually decreased in a predictable manner. Additionally, the degradation behavior of the non-porous and the porous scaffolds were very similar, characterized as bulk hydrolysis. Biocompatibility was also studied, as these materials are intended for eventual use as heart tissue scaffolds. TMC-DLLA materials (1:4 or 1:1 molar ratios) lose their tensile strength in less than five months and are completely resorbed in 11 months. Cell seeding indicated that rat cardiomyocytes attach and proliferate well on the TMC-DLLA co-polymers.

**Figure 4:**
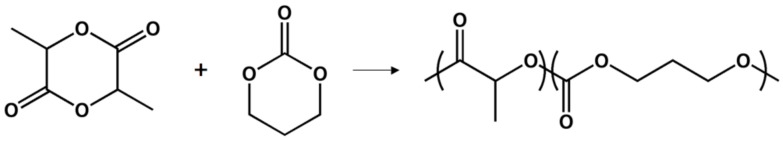
Ring-opening polymerization of d,l-lactide and trimethylene carbonate, resulting in poly(TMC-DLLA).

After extensive *in vitro* testing, transparent poly(TMC-DLLA) samples were evaluated *in vivo* by subcutaneous implantation in rats for up to one year [92]. Although water uptake was low (<2%), the poly(ester carbonate) films were opaque upon surgical retrieval. Previous studies indicated that the integrity of mechanical properties relies on molecular weights of at least 25,000 g/mol [93]. Poly(TMC-DLLA) samples underwent a reduction in molecule weight from 230,000 g/mol to <20,000 g/mol in 12 weeks, *in vivo*. Therefore, applications that necessitate the retention of mechanical strength or rigidity could only be successful for approximately three months. Promisingly, the co-polymer demonstrated excellent tissue tolerance. After surgical insertion, the poly(ester carbonate) film caused a sterile inflammatory reaction and a normal foreign body response, similarly observed after implantation of other biodegradable polymers. A second foreign body response, related to clearance of smaller polymer fragments, was observed that coincided with extensive mass loss. The degradation process of poly(TMC-DLLA) was characterized as being autocatalyzed by bulk hydrolysis, with preference for esters as opposed to carbonates. In total, these films underwent a 96% mass loss in one year.

### Poly(glycerol sebacate)

Current interest in randomly cross-linked polyester networks is due in large part to the pioneering work of Dr. Robert Langer [94]. In 2002, the design of poly(glycerol sebacate) (PGS), a polyester that is analogous to vulcanized rubber, was described. By heating glycerol and sebacic acid for several days, the alcohols and acids reacted to form esters. The pre-polymer form of PGS can be melted or dissolved for further fabrication processes. The design of PGS was motivated by the need for biodegradable materials that are robust in terms of their mechanical properties. It was hypothesized that a strong and biocompatible polyester elastomer could be useful to a wide range of biomedical applications, including tissue engineering and therapeutic delivery. As mentioned above, glycerol and sebacic acid are endogenous to the human metabolism, minimizing the cytotoxic effects due to degradation products. PGS achieved a Young’s modulus, an ultimate tensile stress (UTS), and a rupture strain of 0.282 MPa, >0.5 MPa, and >267%, respectively. *In vitro* biocompatibility was also assessed by seeding fibroblasts onto PGS-coated glass petri dishes. Cells proliferated well, with normal morphologies, when compared to PLGA as a positive control. *In vivo* testing was then performed by subcutaneously implanting PGS films in rats. Based on inflammatory response and the formation of fibrous capsules, PGS implants have less negative physiological consequences than similar PLGA implants. Also, subcutaneous PGS implants were undetectable after 60 days, having been completely resorbed by the rats with no scarring or permanent deformation to the histological structure.

**Figure 5:**
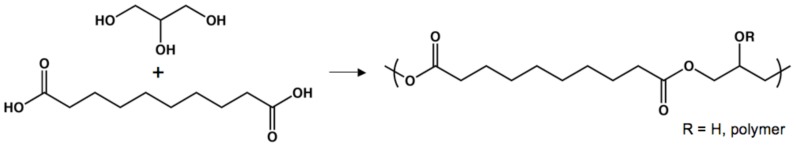
Condensation polymerization of glycerol and sebacic acid leads to a tough polyester elastomer.

A critical aspect of implanted biodegradable materials is how the geometry and mechanical properties of the material change during hydrolysis. PGS was studied throughout the course of *in vivo* degradation in order to determine the timescale during which PGS could be useful [34]. Throughout the 35-day study, PGS segments maintained their overall geometry; PLGA implants, however, were significantly distorted within 14 days. This observation was further characterized by using SEM to visualize the surfaces of PGS and PLGA after implantation. PGS films had similar surface topologies at all time points during the study, while holes and cracks continually developed in PLGA elastomers. The rate of mass loss is also important because mass loss and mechanical integrity are linked. PGS demonstrated a linear rate of mass loss and a linear rate of mechanical strength loss. PLGA samples experienced a dramatic loss in mechanical strength by day 7 and in mass by day 21. These findings are most likely explained by different degradation mechanisms for PGS (surface erosion) and PLGA (bulk degradation). The preliminary results indicate that PGS will be a successful biomaterial, with many *in vivo* applications.

Recently, PGS was used to create scaffolds for myocardial tissue engineering [95]. Designing grafts for myocardial repair has been difficult due to the need to recreate cardiac anisotropy. The vast majority of previously explored materials have been incompatible with the formation of a truly biomimetic system. Taking inspiration from natural collagen fibers that form a honeycomb-like scaffold for cardiac muscle fibers, Engelmayr *et al.* fabricated similar structures composed of PGS. Upon mechanical testing, the stress-strain relationships in the preferred and the cross-preferred directions demonstrated that the films behaved in an anisotropic manner. Further studies were carried out to determine whether PGS honeycomb scaffolds could potentially be used *in vivo* by comparing the flexibility and rigidity of PGS to those of adult rat, right ventricular myocardium. It was determined that both the microfabricated PGS and the natural rat tissue show anisotropic behavior, with similar stress-strain relationships. After simulating physiological loading in a fatigue bath and determining the effect of *in vitro* cell culturing on the anisotropy, PGS honeycomb-like scaffolds were found to be promising, but not yet ideal, materials for myocardial repair. In addition, neonatal rat heart cells, after seeding, oriented in the preferred direction without any external stimuli; this phenomenon is not observed as frequently in isotropic scaffolds. Finally, heart cell contractility could be induced by stimulation with an electric field with directionally dependent electrical excitation thresholds. After conducting their preliminary study, the Langer research group believes that PGS honeycomb-like scaffolds offer a path that can lead to functional myocardium repair through tissue engineering.

### Poly(glycerol sebacate acrylate)

One limitation to the PGS system is that long-term thermal curing could limit encapsulation or application. In an attempt to overcome this challenge, Nijst *et al.* described the design of a photo-curable PGS with tunable mechanical properties and degradation rates [29,96]. By deprotonating free hydroxyl groups and appending acrylate groups, irradiation with UV light yielded elastomeric networks. Additionally, by varying the degree of acrylation, relative to the molar equivalents of free alcohols in the PGS pre-polymer, the mechanical characteristics and degradation rates of the resulting materials could be finely tuned. The Young’s modulus, UTS, and rupture strain ranged from 0.05 to 1.38 MPa, 0.05 to 0.50 MPa, and 47 to 170%, respectively. Interestingly, the PGSA pre-polymer could be combined with other acrylated molecules, offering extended control of macromolecular properties. The co-polymerization of PEG-diacrylate with PGSA enabled further control of characteristics such as mechanical strength, water swelling, hydrophobicity, and degradation rates. *In vitro* biocompatibility was determined by observing adhesion and proliferation of primary human foreskin fibroblasts on different PGSA films.

**Figure 6:**
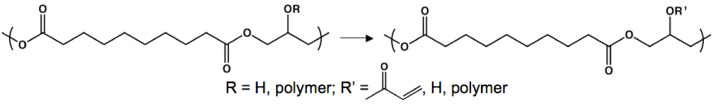
Modification of poly(glycerol sebacate) pre-polymers with acrylate moieties for photo-initiated curing.

The Langer research group very quickly found a practical and inventive application for the photo-curable PGSA. There is a need for strong biodegradable adhesives that can remain attached to tissue while not undergoing permanent deformation when faced with stress. Such a material could be used to replace sutures and staples or to aid in the treatment of ulcers and burns. There seemed to be great potential for PGSA in this regard due to its biocompatibility and the ability to tune its macromolecular properties. In order to design a biodegradable adhesive, Mahdavi *et al.* drew inspiration from the gecko’s ability to adhere to vertical and inverted surfaces [97]. Much research has observed that this phenomenon is based on the micro- and nano-textured feet of geckos, and many examples of gecko-based adhesives have been previously described for material purposes. However, by transferring micro- and nano-scale features to PGSA from etched silicon wafers, gecko-like adhesive tapes aimed at biomedical applications were fabricated. In order to allow the embossed PGSA films to irreversibly bond to tissues in wet conditions (as would be needed *in vivo*), the PGSA surface was functionalized with a carbohydrate-based coating. Oxidized dextran was chosen due to the presence of aldehydes, which serve two purposes: (1) free alcohols can react with oxidized dextran to form hemiacetals, creating a covalent anchor between the carbohydrate chains and PGSA and (2) aldehydes can also react with amines, ubiquitous in biological materials, to form imines, allowing for covalent linkages between the “glue” and tissues. The resulting adhesives combine the bonding forces of gecko feet and covalent bonds. 

As a proof-of-concept, the covalent modification of amine-functionalized glass with oxidized dextran was tested. X-ray photoelectron spectroscopy of the surfaces verified the formation of imine bonds. Additionally, after allowing the carbohydrate derivative and the amine-modified glass to react, the oxidized dextran could not be removed by rinsing the surface. Non-oxidized dextran could be completely removed from the surface in a similar experiment, demonstrating the role of the aldehydes groups in immobilization. Furthermore, both *in vitro* and *in vivo* adhesion to tissue was studied. In addition to being biocompatible, the embossed PGSA films that were coated in oxidized dextran demonstrated significantly more adhesion than flat surfaces and films that were not coated with oxidized dextran. Additionally, tissue adhesion could be optimized by varying the dimensions of the features on the embossed PGSA films, allowing for another level of control over interfacial adhesion. Aiming to surpass the initial success of this preliminary investigation, current research is attempting to design materials for organ-specific applications that may be able to delivery drugs in addition to acting as an adhesive.

### Poly(polyol sebacate)

Sebacic acid has also been polymerized with various sugar alcohols containing at least four hydroxyl groups, allowing for further control of the macromolecular properties of materials based on sebacic acid [98,99]. While all of the sugar alcohols are non-toxic, two of these polyols (xylitol and sorbitol) are endogenous to the human metabolism. Sugar alcohols are ideal for thermal polycondensation because, upon heating or melting, they do not brown the way that sugars can. The polymerization strategy was very similar to the above-mentioned polyester thermosets. Two different poly(xylitol sebacate) (PXS) materials were synthesized, with xylitol-to-sebacic-acid molar ratios of 1:1 and 1:2. By altering the equivalents of the diacid, the mechanical and physical properties could be tuned. For PXS 1:1 [2:1], the Young’s modulus, UTS, and rupture strain was 0.82 MPa [5.33 MPa], 0.61 MPa [1.43 MPa], and 205% [33%], respectively. Additionally, the water swelling of these polymers could also be altered, with values of 5.1% for the 1:1 material and 1.7% for the 2:1 polyester. Both stoichiometric monomer ratios yielded elastomers that exhibited both *in vitro* and *in vivo* biocompatibility, relative to PLGA. After subcutaneous implantation, PXS 1:1 fully degraded in seven weeks, while ~ 75% of PXS 1:2 remained after 28 weeks.

**Figure 7:**
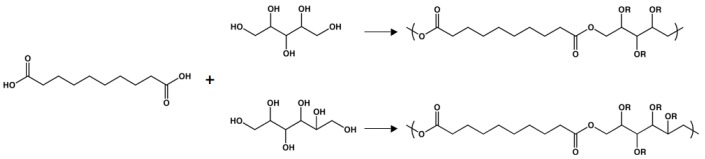
Synthesis of poly(polyol sebacate) using the sugar alcohols xylitol and sorbitol.

By replacing xylitol with sorbitol, the synthesis of poly(sorbitol sebacate) (PSS) was easily achieved [99]. Rather than simply altering the molar ratio of the monomers to tune the mechanical characteristics of the elastomers, the curing conditions were also varied. PSS 1:1 was cured at 120 °C, 2 Pa, for 4 d while PSS 1:2 was cross-linked at 120 °C, 2 Pa, for 5 d. The former demonstrated a Young’s modulus of 0.37 MPa, a UTS of 0.57 MPa, and a rupture strain of 192%. By increasing the molar equivalents of sebacic acid, the mechanical properties were increased, similar to PXS. PSS 1:2 had a Young’s modulus of 2.67 MPa, a UTS of 1.16 MPa, and a rupture strain of 66%. During a 15-week *in vitro* degradation study (PBS, 37 °C), PSS 1:1 and 1:2 lost approximately 15% and 5% of their original mass, respectively. Similar to the previously described materials, PSS 1:1 and 1:2 had acceptable biocompatibility (compared to PLGA standards) based on *in vitro* cell seeding and *in vivo* implantation, suggesting poly(polyol sebacate) as a promising class of polyester elastomers for biomedical applications.

### Poly(amino alcohol sebacate)

After designing PGS and PGSA, the Langer research group focused on making elastomeric materials that exhibited longer *in vivo* degradation rates. This goal was accomplished by including hydrolytically stable amide bonds throughout the polyester matrix [100]. The thermal polycondensation of sebacic acid, 1,3-diamino-2-hydroxypropane, and either glycerol or D,L-threitol led to the synthesis of poly(1,3-diamino-2-hydroxypropane–*co*–polyol sebacate) (APS) composed of four non-toxic monomers [101]. The Young’s moduli, UTS, and rupture strain range from 1.45–4.34 MPa, 0.24–1.69 MPa, and 21–92%, respectively. Additionally, *in vitro* degradation studies were conducted in a sodium acetate buffer with a pH of 5.2. After incubating in the degradation solutions, APS materials demonstrated hydrolytic degradation of 42.8%–97.0%. In addition to extended degradation profiles, post-polymerization modifications could potentially be achieved through functionalization reactions aimed at the free amines. Overall, these materials were biocompatible in an *in vitro* and an *in vivo* setting, with projected *in vivo* degradation half-lives of up to 20 months. In terms of macrophage concentration and fibrous capsule formation observed in subcutaneous implants, APS demonstrated milder foreign body response than PLGA.

**Figure 8:**
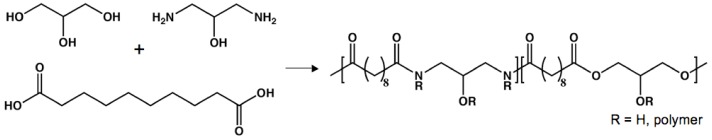
Synthesis of poly(amino ester) elastomers from sebacic acid, glycerol, and 1,3-diamino-2-hydroxypropane.

### Poly(diol-co-citrate)

In order to create polyester elastomers, the research group of Dr. Guillermo Ameer found an ideal tri-functional monomer in the Krebs cycle. Rather than using a diacid with polyols, the cardinal publication from Dr. Ameer’s group demonstrated polyester elastomers based on citric acid, which includes three acid groups and one alcohol [102,103]. Initially, these materials were synthesized through thermal polycondensation with aliphatic diols: 1,6-hexanediol, 1,8-octanediol, 1,10-decanediol, and 1,12-dodecanediol. While several diols were tested, Dr. Ameer’s group focused on poly(1,8-octane citrate) (POC). Using an easy fabrication process, non-toxic monomers, and a curing technique that allows for facile tuning of physical and mechanical properties, inexpensive materials that have amazing potential in soft tissue engineering were designed. The curing process simply involves heating the pre-polymer solution, similar to PGS. However, POC was able to form insoluble elastomers at temperatures as low as 37 °C. By adjusting the curing temperature and duration, POC can obtain Young’s moduli ranging from 0.92 MPa to 16.4 MPa, ultimate tensile strengths up to 6.1 MPa, and a maximum of 502% elongation at break. The Young’s modulus and ultimate tensile stress compare well to elastin from bovine ligaments (1.1 MPa and 2 MPa, respectively). 

The applications of POC have been directed towards cardiovascular tissue engineering. Accordingly, the biocompatibility of these materials was evaluated with human aortic smooth muscle cells (HASMCs) and human aortic endothelial cells (HAECs). When compared to the non-toxic poly(l-lactide) (PLLA), POC films are biocompatible with both cell populations. Finally, the *in vivo* reaction of Sprague-Dawley rates to implanted POC films was determined to be excellent; no chronic inflammatory response was observed. Additionally, various types of scaffolds were designed to explore their fabrication potential of POC. The resulting constructs were soft and able to fully recover from deformation. One example of these scaffolds, a biphasic and tubular structure with a non-porous, skin-like interior and a porous exterior, has been used extensively in blood vessel engineering.

**Figure 9:**
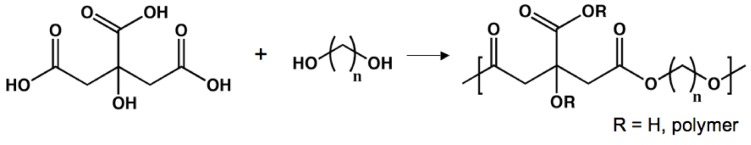
Synthesis of poly(diol-co-citrate) from citric acid and aliphatic diols.

In order to determine whether or not the biphasic tubular POC scaffolds would be appropriate candidates for *in vivo* applications, several basic characteristics were studied, including mechanical properties and biocompatibility [104]. By first observing the implantable poly(diol-co-citrate) (PDC) scaffolds *in vitro* and *in vivo*, potential surgical problems, including compliance mismatch and thrombosis, could be identified and circumvented. It was determined that the mechanical characteristics of the PDC scaffolds match those of native blood vessels well [105]. Varying the diol and the curing conditions, the mechanical properties could easily be tuned. These tubular constructs were able to achieve Young’s moduli of ~1.5 MPa, ultimate tensile stress of ~3 MPa, and rupture elongation of ~350%. Additionally, testing determined the biphasic scaffolds to have a compressive modulus of 0.152 MPa, rendering the materials soft and able to fully recover from deformation. Burst pressure and compliance values (>120 mmHg and 12.7%, respectively) also offer PDC hybrid scaffolds as potential synthetic blood vessels.

*In vitro* testing determined that these materials are biocompatible. Importantly, different types of cells could be seeded to different areas of the scaffold. HASMCs were seeded to the porous exterior phase, while HAECs were cultured on the skin-like, non-porous interior of the artificial blood vessel. This compartmentalization of cells may enable the design of blood vessel scaffolds that very accurately mimic the local environment of native vessels [106]. Finally, *in vivo* biocompatibility was also used to fully understand the biomedical potential of biphasic PDC constructs. Subcutaneously implantation of scaffolds demonstrated significant tissue ingrowth and a foreign body response similar to that of PLLA.

The Ameer group furthered their work in *in vivo* vascular tissue engineering by making hybrid materials of POC and expanded polytetrafluoroethylene (ePTFE) [107]. The current standard-of-care for vascular grafts involves either ePTFE or surface modified ePTFE tubes. While this strategy has proven effective for large-diameter blood vessel applications (>6 mm diameter), ePTFE small-diameter blood vessels have performed less successfully due to complications, including graft occlusion due to blood clots [108,109]. The surface of ePTFE is inherently thrombogenic and the hydrophobic surface can reduce the efficiency with which an endothelial layer of cells can form [108,110,111]. Currently, efforts to minimize thrombosis by enhancing endothelialization has primarily focused on graft coatings, the conjugation of biomolecules, or the immobilization of non-degradable polymers. This process, however, introduces possible coating-based complications due to compliance, biological response, cost, and long-term efficacy. 

Based on the previously described success of bi-phasic tubular POC scaffolds, the Ameer group demonstrated that their polyester acts as a suitable coating for ePTFE vascular grafts. The biocompatibility of the implant was improved without negatively affecting compliance. Additionally, POC-ePTFE grafts caused reduced platelet adhesion, aggregation, and activation, when compared to control ePTFE grafts. To conclude the study of POC-ePTFE grafts, *in vivo* testing was performed to allow access to surgically relevant data. Both the number of macrophages present and the thickness of the fibrin coagulum were significantly reduced for the POC-ePTFE graft when compared to the ePTFE control. Collectively, the finding indicated that POC has a very promising future in surgical repair of blood vessel.

In order to further understand the potential of POC in vascular surgery, fully biodegradable vascular scaffolds were prepared [112]. When compared to the previous study, the difference is that that ePTFE graft was replaced with a non-porous POC tube, creating a graft fabricated fully from the polyester. Initially, platelet adhesion and activation was studied on glass, ePFTE, PLGA, and POC. Not only do fewer platelets attach to POC than any of the other materials, the morphology of the platelets is very spherical. When platelets attach to a surface and adopt extended, spread morphologies, the biological result is aggregation and activation, which leads to clotting [113,114]. The anti-thrombogenic properties of the completely degradable POC graft make it an excellent candidate for blood vessel repair. Also, the plasma recalcification kinetics are significantly lower for POC, relative to the other materials. The coagulative properties of POC are much more suitable for contact with blood than tissue culture plastic and PLGA. Another critical biological process that affects implants is the inflammatory response. In order to monitor the inflammatory response of POC *in vitro*, the activation of human THP-1 cells (an alternative to problematic peripheral blood monocytes) was monitored based on changes in the level of IL-1β and TNF-α [115,116]. All of the materials, including POC, cause THP-1 cells to produce very low levels of activation markers, when compared to the positive control (lipopolysaccharide). Relative to positive controls, POC had satisfactory hemolysis and protein adsorption values, comparable to PLGA, ePTFE, and tissue culture plastic. Finally, HAECs were seeded onto POC scaffolds under flowing conditions. While approximately 100% cells attached to glass surfaces, only 69% were retained on POC constructs. However, while cell attachment values were non-ideal, over half of HAECs were able to remain on POC under physiological shear stress conditions, a promising property. All of the above results are encouraging, suggesting that POC is an excellent polyester candidate for *in vivo* vascular tissue engineering.

### Dendritic Poly(glycerol succinate)

The majority of the previously described research has all focused on randomly cross-linked polyester elastomers. However, other classes of polyester tissue scaffolds have also been studied [117]. For example, the Grinstaff lab has designed biodegradable dendrimers composed of succinic acid and glycerol moieties [118,119,120]. The materials are entirely composed of natural human metabolites and, therefore, complete hydrolytic degradation should not lead to the production of any toxic compounds. By using benzylidene acetal protecting groups, stepwise synthesis led to the design of monodisperse dendrimers (PDI = 1.02) where the terminal functionality could be controlled (either acids or alcohols). The degradation kinetics were tuned according to hydrophobicity of the monomers, the dendrimer generation, and the inherent reactivity of the materials. 

**Figure 10:**
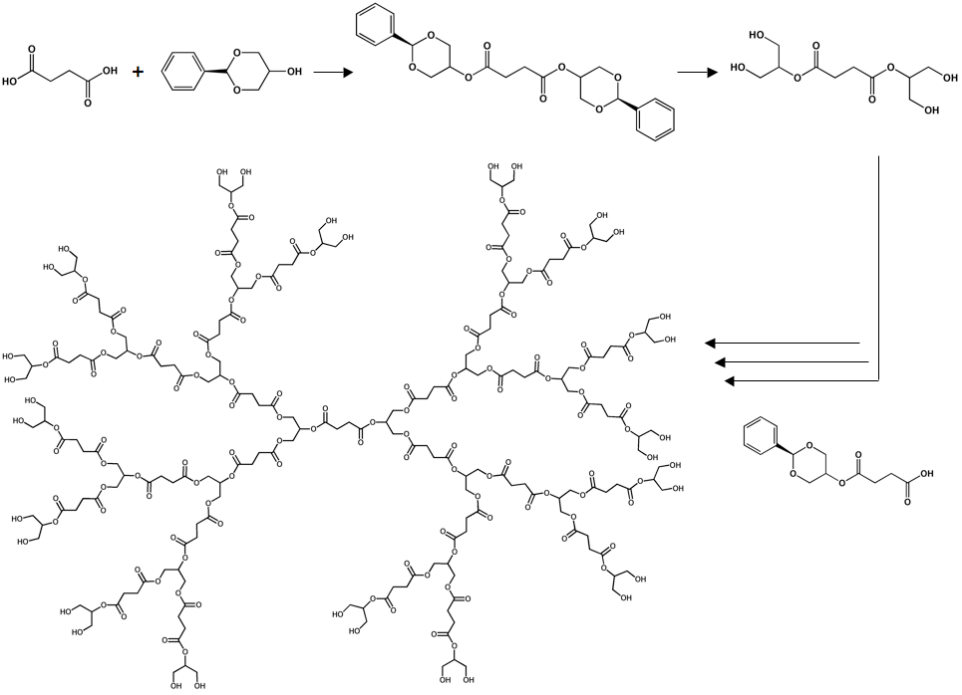
Design of dendrimers composed of succinic acid and glycerol.

In order to create appropriate materials for tissue engineering, photo-crosslinkable dendrimers were designed by appending methacrylate moieties to the terminal alcohols. The Grinstaff group has utilized their materials to repair corneal wounds that are usually repaired with sutures. Due to drawbacks related to suturing, research in the Grinstaff lab has focused on the delivery and curing of liquid pre-polymers to seal corneal wounds *in situ*. The medical adhesive that has been characterized most fully is similar to the product shown in Figure 10, except that the core is based on poly(ethylene glycol) (PEG) instead of succinic acid. A first generation, methacrylated dendrimer was delivered to a corneal laceration and cross-linked so that the leak pressure could be determined. Standard nylon sutures obtain a leak pressure 90 mm Hg, while the photo-cured polyester could withstand pressures of up to 171 mmHg. Both of these values are well above the physiological intraocular pressure of 15-20 mmHg [121]. In addition to having a larger leak pressure, the dendrimer adhesive, without causing additional trauma, allows for a recovery period that is five times faster than recovery from sutures.

In addition to repairing corneal lacerations, the Grinstaff lab has used their photo-curable polyester dendrimers to secure laser-assisted *in situ* keratomileusis (LASIK) flaps [122]. LASIK is the popular surgical procedure that corrects myopia and astigmatism by moving a corneal flap, using a laser to ablate the cornea stromal tissue, and repositioning the corneal flap. Commonly, sutures are used to secure the LASIK flaps during the recovery period. However, as previously stated, sutures are not the ideal method for this procedure due to the extra trauma that is inflicted upon the corneal tissue. Using donated human eyes, the LASIK flap was secured by curing the PEG-based dendrimer with an argon laser. After cross-linking the adhesive, the flaps were manually tested to see the strength of the seal. Despite considerable force, the dendrimer seal remained intact. Although these results are qualitative, the poly(glycerol succinate) dendrimers have a large amount of potential dealing with surgical procedures focusing on cornea repair.

### Poly[tetra(ethylene glycol) α-ketoglutarate]

Another metabolite from the Krebs cycle, αKA, was the focus of research in 2008 from the Yousaf group [123]. Combining αKA and tetra(ethylene glycol), linear polyketoester chains were biocatalytically synthesized with Lipase B (from *Candida antarctica*), an enzyme that is able to withstand the high temperatures needed for polycondensation (Figure 8) [124]. Selecting an ethylene glycol oligomer as the diol resulted in completely amorphous polymer chains. However, the location of the ketone immediately adjacent to the acid in αKA leads to formation of very short polyester chains. Molecular weights of M_n_ = 1,200 g/mol were the largest that could be achieved. Also, αKA is incompatible with metal catalysts that are commonly used for polyester synthesis. 

**Figure 11:**
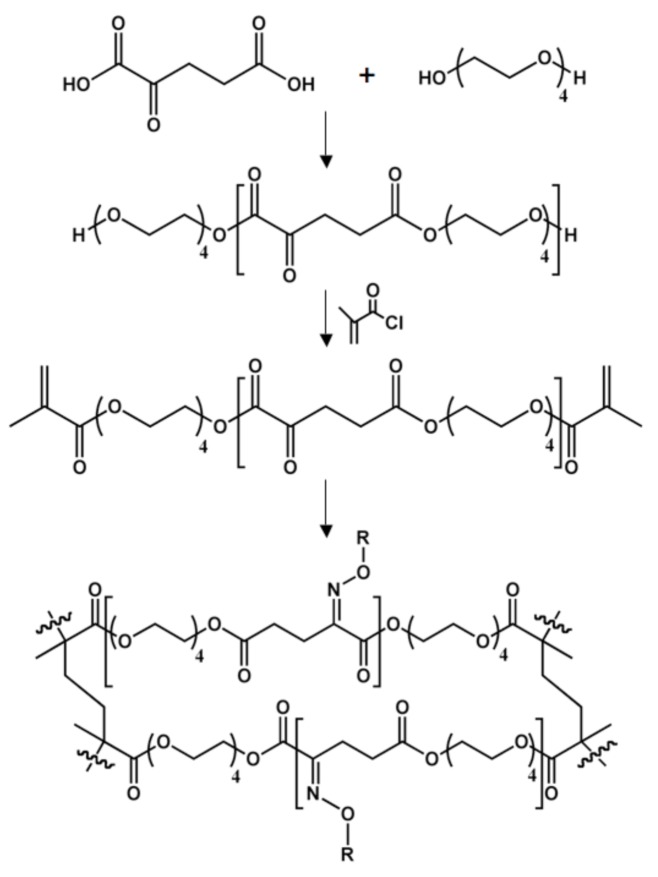
Synthesis of chemoselective elastomers based on α-ketoglutaric acid and tetra(ethylene glycol). Oxime formation occurs from the reaction of the ketone-containing materials with oxyamine-terminated ligands.

The presence of a ketone in the repeat unit allow for chemoselective immobilization of oxyamine-containing molecules. Oxime formation is fast, very specific, compatible with organic or aqueous environments, hydrolytically stable, and proceeds at physiological conditions [125,126,127,128,129,130]. These characteristics make the reaction between ketones and oxyamines an ideal strategy for the modification of biomaterials. This immobilization strategy was especially attractive because of how easily oxyamines can be introduced into an extremely wide range of materials, such as carbohydrates, peptides, proteins, pseudo-lipids, and therapeutic agents [130].

In order to create polyketoester elastomers, the linear chains were capped with methacrylate groups, allowing for photo-initiated curing. After cross-linking with UV light, the resulting films were biologically inert due to the high content of tetra(ethylene glycol) moieties [131,132]. Without further modification, cells were unable to attach to the polyketoester films. However, upon reaction with an oxyamine-terminated RGD peptide (the minimal amino acid sequence required to interact with integrins, a family of cell surface receptors), cells were able to attach, proliferate, and migrate [133,134]. While receiving promising results with poly[tetra(ethylene glycol) α-ketoglutarate], the authors decided to progress to randomly cross-linked polyester elastomers based on αKA similar to those described above.

### Poly(triol α-ketoglutarate)

Thermal polycondensation has also been used to achieve elastomeric films from αKA. Previously reported, polymerization of α-ketoglutaric acid and one of three triols (glycerol, 1,2,4-butanetriol, or 1,2,6-hexanetriol) led to the synthesis of poly(triol α-ketoglutarate) (PTK), polyester thermosets that contain ketones [135]. Varying the triol, the curing temperature, and/or the duration of the curing process allowed for a wide range of mechanical properties and degradation rates. The values of the Young’s modulus (0.1–657.4 MPa), ultimate stress (0.2–30.8 MPa), and ultimate strain (22–583%) encompass the mechanical properties of many biological materials, increasing the probability of success for the use of poly(triol α-ketoglutarate) as a biomaterial [10,11]. Additionally, the PTK series hydrolytically degraded in as fast as two days and as long as 28 days in phosphate-buffered saline solutions at 37 °C. In general, all of the PTK series degrades very rapidly. This occurs due to the presence of the ketone immediately adjacent to an ester. Acting as an electron-withdrawing group, the ketone increases the reactivity of the ester carbon, rendering it more susceptible to hydrolysis.

For post-polymerization modifications, the repeat units contain ketones, which are capable of reacting with a variety of oxyamine-terminated molecules to generate stable oxime linkages. To demonstrate oxime formation, a solution of an oxyamine-modified dye was flowed through a microfluidic cassette onto the surface of PTK films [135]. After rinsing the polyketoester film, the pattern is clearly visible using fluorescence microscopy. Oxyamine-conjugation will be used in the future to design tissue scaffolds that are functionalized with biologically relevant ligands.

Finally, as future applications will focus on the biomedical fields, the biocompatibility of poly(glycerol α-ketoglutarate) (PGK), poly(1,2,4-butanetriol α-ketoglutarate) (PBK), and poly(1,2,6-hexanetriol α-ketoglutarate) (PHK) was studied. As all three materials contain the metabolite αKA, the cytotoxicity was expected to be low. Based on cell proliferation and morphology, all materials were deemed biocompatible.

Because the PTK series of polyester elastomers degrades rapidly in PBS at 37 °C, we believed that dilute solutions of NaOH could cause accelerated hydrolysis [136]. After observing that this hypothesis was correct, local degradation was harnessed by using microfluidics. The delivery of NaOH solutions in micro-channels caused controlled hydrolysis that led to the formation of etched features. This technique is very straightforward, flexible, and inexpensive. Because the polymers are made from αKA, a ketone is present in the repeat unit. Microfluidics can also be used to deliver oxyamine-containing ligands in a controlled manner. As etching and functionalization are compatible with one another, we were also able to create functionalized micro-channels in the PTK films. Finally, by sealing an etched film to a flat piece of PTK, an enclosed and biodegradable microfluidic system can be fabricated. This was demonstrated by flowing an ethanolic solution of ink through the device, enabling easy visualization of the features within the polyketoester matrix. Future studies aim to create these microfluidic devices in order to explore bioreactors and synthetic vasculature.

**Figure 12:**
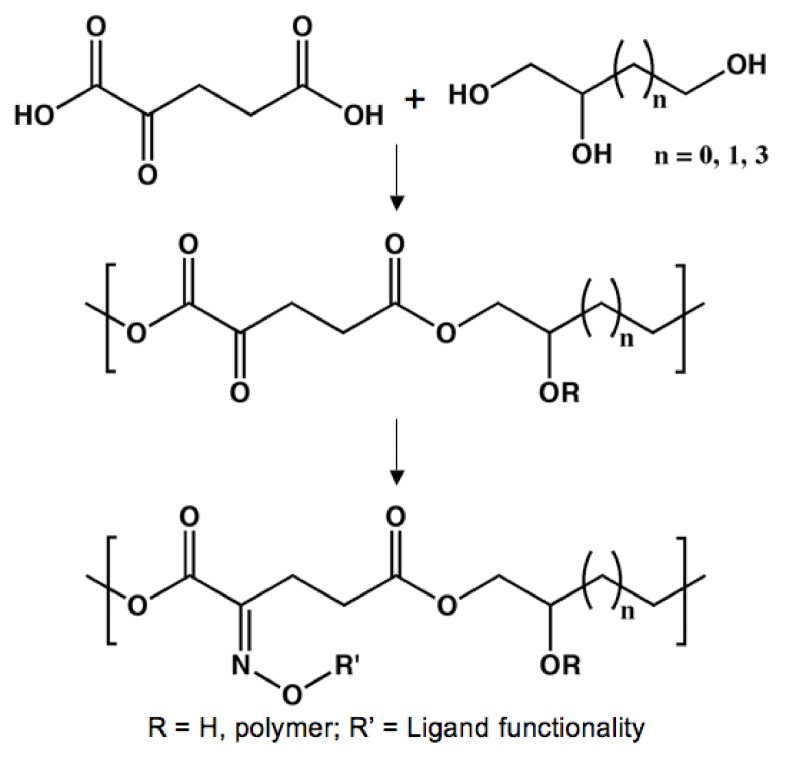
Synthesis of poly(triol α-ketoglutarate) thermosets containing ketones for oxime-based functionalization.

### Poly(propylene fumarate)

Since its introduction a decade ago, poly(propylene fumarate) (PPF) has quickly become a commonly studied scaffold material [137]. By basing the design on two resorbable monomers, the degradation products of PPF are non-cytotoxic. Similar to many of the materials synthesized from ring-opening polymerizations, the macromolecular properties of PPF are very dependant upon the methods used for synthesis, cross-linking, and fabrication [138,139,140,141]. In order to determine the appropriate application towards which PPF should be applied, the *in vitro* degradation rates were studied. So that a wide range of properties could be observed, bulk and different porosity (70% and 80%) materials were observed. As expected, the hydrolytic degradation rate was a function of both the porosity and the size of the initial porogen. The fastest degrading materials had more and larger pores. The study lasted 32 weeks, allowing time for substantial hydrolysis to occur. The amount of mass lost for PPF degradation ranged from 15 % – 30 %. After noting these degradation rates, the most common application for PPF has been tissue engineering of bone. Bone was a logical medium due to the hydrophobicity of PPF and the high degree of potential cross-linking with an alkene in each repeat unit.

**Figure 13:**
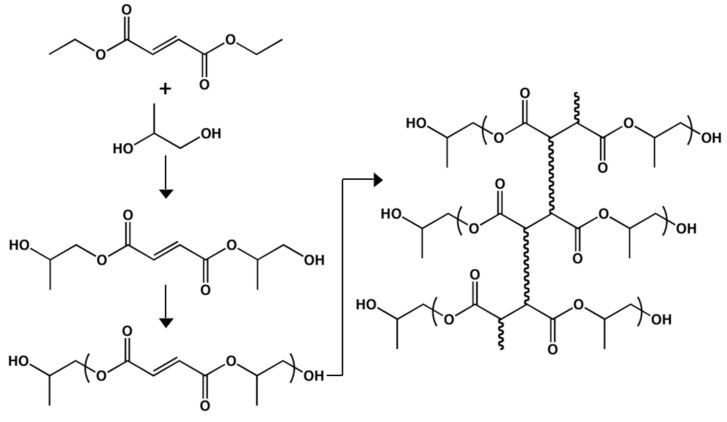
Synthesis of poly(propylene fumarate) containing alkenes as cross-linking sites.

As bone exists near soft and hard tissues *in vivo*, PPF was studied in animal models to ensure biocompatibility when interacting with both [142]. For the live studies, rabbits were used as the host animal. In order to investigate PPF-soft tissue interactions, samples were subcutaneously implanted. Photo-cured PPF has great potential in tissue engineering, based on its interactions with soft tissue. A fibrous capsule surrounding the scaffolds was noted. However, the thickness of the cellular encapsulation decreased with time, implying that the rabbit tissue found the implant and its degradation products to be acceptable. The porosity of the PPF films increased with time, which was expected due to the surface degradation of the polyester backbone throughout the study. PPF was also studied as a bone substitute so that its interactions with hard tissue could be investigated. After creating four non-fatal cranial defects in the rabbits, the polyester was inserted. The hard tissue response was similar to the interaction of soft tissues with PPF. While the scaffolds were found to contain low amounts of bone ingrowth, 40% of the scaffolds exhibited direct bone-implant contact. While other materials have performed similarly, PPF may be differentiated due to the minimal thickness of the fibrous capsule and the slower degradation rate [143,144,145,146]. Based on these results, PPF has been considered a promising option for tissue engineering of bone.

## Conclusions

These materials offer a number of potential applications throughout the biomedical fields. While many uses have been researched, the application that has been most extensively studied is cardiovascular tissue engineering. The lactide/ε-caprolactone co-polymer has currently produced the most success, as it has been clinically applied. Several human patients were given polyester autografts with no side effects exhibited. None of the other materials discussed here have progressed as far, in terms of *in vivo* success. However, while the poly(diol-citrate) series has not been applied to human models, it has been implanted in animal models for several different studies. Instead of designing artificial arteries, the Ameer research group has shown innovative work in the field of blood vessel engineering. The unique design of their biphasic scaffold has allowed for compartmentalization of different cell types, enabling the fabrication of truly biomimetic scaffolds. Cardiovascular tissue engineering has also become a goal of the Langer lab, with the introduction of their honeycomb-like films that maintain cardiac anisotropy. 

While tissue scaffolds cardiovascular applications have been most studied, the key observation is that a wide range of polyesters can be designed from monomers that are endogenous to the human metabolism. The hydrophobicity, biocompatibility, and mechanical properties span a range that encompasses the properties of many biological samples. A large number of these polyesters have been synthesized in a manner that has focused on maximizing biocompatibility, assuming that cytotoxicity would be minimized by using natural metabolites as monomers. Another characteristic that has received considerable attention is the relationship between the mechanical properties of the polyester and those of the original tissue. By matching the strength and flexibility of the soft tissue, a scaffold has the highest probability of success *in vivo*. To date, the previously described materials based on monomers endogenous to the human metabolism have achieved Young’s moduli between 0.048 MPa and 657.4 MPa, UTS values between 0.054 MPa and 53.0 MPa, and rupture strain values between 4% and 854.4%. Many of these polyesters have been described as biocompatible based on *in vitro* and/or *in vivo* testing. In the future, as more tissue scaffolds are designed and existing materials are further characterized, the probability of successfully using human metabolites to create scaffolds for tissue engineering should continue to increase.

## Supplementary Files

Supplementary File 1
