# Supplementary material for: Design and Applications of Biodegradable Polyester Tissue Scaffolds Based on Endogenous Monomers Found in Human Metabolism
Source: Molecules. 2009 Oct 12;14(10):4022–50. doi: 10.3390/molecules14104022 (PMC6255442; doi:10.3390/molecules14104022)
Supplement: Supplementary File 1 [file molecules-14-04022-s001.pdf]

Molecules **2011**, *16*

OPEN ACCESS

molecules

ISSN 1420-3049

www.mdpi.com/journal/molecules

Correction

Barrett *et al*. Design and Applications of Biodegradable Polyester Tissue Scaffolds Based on Endogenous Monomers Found in Human Metabolism. *Molecules* 2009, *14*, 4022-4050

Devin G. Barrett and Muhammad N. Yousaf *

Department of Chemistry and Carolina Center for Genome Science, University of North Carolina at Chapel Hill, Chapel Hill, NC 27599, USA

* Author to whom correspondence should be addressed; E-Mail: mnyousaf@email.unc.edu.

Received: 17 May 2011 / Published: 23 May 2011

The authors wish to make the following correction to this paper [1]:

The correct journal name for reference [2] is *Tissue Engineering*, therefore this reference is hereby corrected to read as follows:

2. Yang, S.; Leong, K.F.; Du, Z.; Chua, C.K. The design of scaffolds for use in tissue engineering. Part I. Traditional factors. *Tissue Eng.* **2001**, *7*, 679-689.

Reference

1. Barrett, D.G.; Yousaf, M.N. Design and Applications of Biodegradable Polyester Tissue Scaffolds Based on Endogenous Monomers Found in Human Metabolism. *Molecules* **2009**, *14*, 4022-4050.

© 2011 by the authors; licensee MDPI, Basel, Switzerland. This article is an open access article distributed under the terms and conditions of the Creative Commons Attribution license (http://creativecommons.org/licenses/by/3.0/).
